# Understanding and supporting the mental health and professional quality of life of academic mental health researchers: results from a cross-sectional survey

**DOI:** 10.1186/s12889-025-21823-3

**Published:** 2025-02-15

**Authors:** Jacks Bennett, Nina Di Cara, Lizzy Winstone

**Affiliations:** 1 Population Health Sciences, Bristol Medical School, Canynge Hall, 39 Whatley Road, Bristol, BS8 2PS UK; 2 School of Psychological Science, University of Bristol, 12a Priory Road, Bristol, BS8 1TU UK

**Keywords:** Researcher mental health, Professional quality of life, Lived experience, Mental health, Secondary traumatic stress, Burnout, Compassion satisfaction, Coping strategies, Higher education, Workplace support

## Abstract

**Background:**

Academic mental health research is critical to understanding, treating and preventing poor mental health. Researchers often have their own lived experience of a mental health condition, but despite potential exposure to distressing research material, the mental health and work-related quality of life of mental health researchers is not systematically supported in UK universities. This study aimed to quantitatively characterise the mental health experiences, professional quality of life and workplace support needs of this group.

**Methods:**

UK academic mental health researchers (*n* = 254) answered an online survey in March 2024. Using linear regression modelling, we tested associations between socio-demographic, mental and physical health and work-related factors alongside negative and positive aspects of professional quality of life, i.e., secondary traumatic stress, burnout and compassion satisfaction, as well as maladaptive coping strategies such as alcohol and drug use or sickness absence from work. We also compared researchers’ workplace support experience with their perceived support need and examined implications for funding applications.

**Results:**

Having personal lived experience of a mental health condition showed the strongest association with poorer professional quality of life outcomes. Mental health researchers using qualitative methods also reported higher levels of secondary traumatic stress and burnout than those using quantitative methods, as did those with a disability or chronic illness (compared to those without). Researchers with personal lived experience of a mental health condition also showed ten times the odds of taking sickness leave to cope with work related feelings. There were important differences between the types of workplace support researchers experience with those they report needing. Our evidence also points to more guidance needed on factoring support into research projects.

**Conclusions:**

Our findings highlight the wealth of lived experience amongst mental health researchers, and the importance of providing systematic proactive support for this group, as well as for those with a disability or chronic illness, or those using qualitative methodologies. With sickness absence having considerable economic and organisational consequences for employers and funders, recommendations include developing researcher well-being plans, regular end of project debriefs, development and training on challenging topics, and clearer consideration of researcher support in funding applications.

**Supplementary Information:**

The online version contains supplementary material available at 10.1186/s12889-025-21823-3.

## Introduction

The World Health Organisation (WHO) defines mental health as ‘a state of mental well-being that enables people to cope with the stresses of life, realise their abilities, learn well and work well, and contribute to their community’ [1]. It suggests that mental health is experienced on a continuum, is more than an absence of mental health conditions and is influenced by several factors. The WHO point to work-related mental health as a growing area of importance [1]. Poor mental health, including mental health conditions, suicide and self-harm, represents a global health, social and economic burden [2, 3]. Mental health research is critical to the understanding and prevention of mental health conditions, as well as informing the development of effective treatments and policies, reducing stigma, and promoting good mental health and well-being across society [4]. The field of mental health research spans a wide range of academic disciplines, including public health, epidemiology, psychiatry, psychology, sociology, medical anthropology, data science and beyond.

The nature of topics encountered in the field of mental health research mean that researchers are likely to face distressing material in the course of their work [5, 6]. This might include reading graphic case reports or autopsies, literature and statistics relating to emotionally challenging topics, or having conversations with vulnerable participants in distress. While research ethics committees have improved ethical procedures to protect study participants in recent decades, there has not been equivalent consideration of the impact on researchers working on sensitive data, many of whom also have their own ‘lived experience’ of the topic they work on [5, 7, 8]. In parallel, there has been a growing emphasis on employing academic researchers with lived experience, who can be considered ‘dually qualified people who have both academic credentials and experiential expertise’ [9].

Working with emotionally challenging material has potential consequences for professional quality of life and other work-related mental health outcomes [7]. As a construct, professional quality of life in helping and caring professions has been shown to reflect three main dimensions: secondary traumatic stress (STS), burnout, and compassion satisfaction [10, 11]. Secondary – or vicarious – traumatic stress can arise as a result of the indirect exposure to traumatic experiences, and is considered a form of occupational stress [12, 13]. Symptoms of STS can resemble those of Post-traumatic stress disorder (PTSD), including intrusive thoughts and feelings of helplessness or fear. These effects can be upsetting, disruptive and enduring [12–14]. The related phenomenon - burnout - can also result from prolonged exposure to stress, and presents as a state of physical, emotional, and mental exhaustion [15]. Secondary traumatic stress and burnout – sometimes referred to together as compassion fatigue – are prevalent among those in helping or caring professions, including child protection and social care [16], emergency responders [17], and intensive care nurses [18]. Compassion satisfaction is a positive dimension of professional quality of life, referring to the sense of fulfilment derived from care-giving roles and helping others [10, 11]. High levels of compassion satisfaction are thought to mitigate against the development of STS and burnout [19]. 

Whilst secondary traumatic stress is well-evidenced in those helping or caring for people experiencing trauma or poor physical or mental health [19], less is known about the experiences of researchers who work with emotionally challenging material, for example relating to topics such as self-harm, suicide or poor mental health. Williamson et al. [12] suggest that while STS is thought to be most relevant to those researchers with lived experience of the condition or situation under study, it can affect any researcher exposed to graphic or distressing detail within research data. Burnout can also affect any researcher, although it may not necessarily relate to mental health topics under investigation (for example, resulting instead from precarious employment contracts common within the higher education sector) [12, 20]. STS and burnout have implications not just for the researcher – emotional exhaustion, anger, depersonalisation or cynicism towards participants, decreased empathy, decreased job satisfaction – but also for their employing organisations, in the form of sickness absence and decreased quality of work [12–15]. 

Qualitative evidence from other fields suggests that, whilst researchers working on emotionally challenging topics such as gender-based violence, find their work rewarding and inspiring, they are simultaneously vulnerable to STS and burnout, including over-identification with individual stories or becoming desensitised to shocking material [12, 21]. An autoethnographic study exploring the experiences of ten researchers working on a gender-based violence project identified distress arising from feelings of ongoing concern with individual participants and a frustration at being unable to ‘make things better’ for vulnerable participants [12]. In a similar case study, interviews with eight research assistants on a suicide prevention trial in New Zealand highlighted researcher STS relating to processing case-note data that included graphic descriptions of self-harm injuries, and detailed histories of abuse [22]. Themes identified from these interviews included distress relating to the level of detail within the data, being drawn into and dwelling on individual stories, emotional exhaustion and compassion fatigue related to a cumulative effect of long study periods involving repeated exposure to distressing material, becoming sensitised to the possibility of harm, and the need for researchers to develop their own coping strategies because of a lack of formal support as standard. A lack of clinical training for research assistants was perceived to have led to them feeling unprepared for the emotional impact of this in-depth research [22]. 

Evidence suggests that women are more susceptible to STS and burnout compared to men [23, 24], and that researchers from minoritised groups, including LGTBQIA + and those with limiting long-term illness or disability, may be particularly vulnerable to poorer outcomes due to underrepresentation or studying topics linked to their own experiences of prejudice [7, 24]. Studies of helping or caring professions have found all three sub-dimensions of professional quality of life (STS, burnout and low compassion satisfaction) to be associated with poor mental health, including sleep disturbances, intrusive thoughts, anxiety, depression, and maladaptive coping strategies such as alcohol and substance use [19, 23, 25–28]. A recent study of health professionals during the Covid-19 pandemic also found a clear association between increased psychological distress (anxiety, depression and stress) and STS and burnout, over and above any stress related to the pandemic [28]. 

Academic mental health researchers in non-clinical roles do not receive support for their own mental health and well-being as standard. Despite dealing with many of the same emotionally challenging topics, this lack of systematic support differs to those working in clinical or therapeutic roles, who are required to engage in clinical supervision and reflective practice, developing strategies for processing emotions resulting from their work [12]. Reflective practice can reduce the risk of STS or burnout by providing space to process work-related emotional demands, identifying early signs of stress and developing coping strategies, and encouraging self-care [12]. In research it is likely that more ‘junior’ staff members will be those directly conducting interviews, data analysis and participant facing tasks. These, combined with the lack of control associated with being a researcher employed on a project rather than being the Principal Investigator (PI), are thought to be risk factors for poorer professional quality of life outcomes among early career researchers in particular [21, 22, 24]. Understanding the factors influencing professional quality of life among mental health researchers is not only essential for protecting researcher mental health and resilience but has implications for productivity and quality of research, empathy for research participants, research culture within Higher Education Institutions (HEIs) and organisational policy regarding researcher support and professional development requirements [24]. 

Several studies have now qualitatively explored experiences of secondary trauma and burnout among academic researchers working on distressing topics in other fields [12, 21, 22, 29]. These studies have identified important themes related to researchers’ professional quality of life in academic settings, including emotional demands of the research relating to sensitive or upsetting topics, levels of detail within research data, prior or current trauma, long-term exposure to distressing topics, mental health impacts, self-care and institutional support services. Yet there remains a lack of quantitative evidence for risk and protective factors for maintaining a positive professional quality of life among academic researchers working on topics relating to mental health.

Interviews with trauma researchers as part of a UK Researcher Wellbeing Project have also identified several informal coping strategies for managing feelings arising from work on emotionally challenging topics [21]. Skinner et al. found these include effective strategies such as exercise, hobbies, maintaining hope and a sense of purpose in work, and working only office hours and weekdays. However, coping strategies which may be considered harmful were also reported, with some participants highlighting the use of alcohol to numb their emotions or manage stress [21]. Alcohol (mis)use has been identified by others as an evidence gap in the literature on work-related quality of life and informal coping strategies [19]. The use of sick leave as an informal coping strategy has also been understudied, though there is emerging evidence to suggest associations between burnout and work absenteeism in clinical professions [25, 30]. Despite recent efforts like the Researcher Wellbeing Project, to develop recommendations for supporting academic researchers working on sensitive issues, there is still a lack of empirical evidence for the types of formal workplace support already in place UK higher education institutions (HEIs), or the informal coping strategies researchers use to alleviate any impact of their work [15, 21, 24, 29]. 

This study will address the research gaps identified above by surveying mental health researchers across the UK, using a validated professional quality of life scale to examine levels of STS, burnout and compassion satisfaction, alongside a range of mental health experience, informal coping and workplace support questions developed from previous qualitative research findings [10, 11, 20, 21, 29]. 

## Aims, hypotheses and research questions

The overall aims of this study were to (i) characterise the self-reported mental health experiences of academic mental health researchers in the United Kingdom (UK), and (ii) to examine professional quality of life, informal coping strategies and workplace support needs of this group. We tested the following confirmatory and exploratory hypotheses and research questions based on existing qualitative research, existing evidence for vulnerabilities in certain groups, and in line with a set of pre-registered aims (https://osf.io/v2qwd/) [7, 12, 21, 23, 24].

### Confirmatory hypotheses

H1. Mental health researchers will be more likely to experience higher secondary traumatic stress than their peers if they are (i) early career; (ii) use qualitative methods; (iii) have personal lived experience of a mental health condition; (iv) have a physical disability or chronic illness; (v) have caring responsibilities; (vi) are not in secure employment; or (vii) are from minoritised groups.

H2. Mental health researchers will be less likely to experience secondary traumatic stress if they have professional training in reflective practice or in managing their own well-being.

### Exploratory hypotheses

H3. There will be a difference in burnout among mental health researchers according to the following factors (i) being early career; (ii) using qualitative methods; (iii) having personal experience of a mental health condition; (iv) having a physical disability or chronic illness; (v) having caring responsibilities; (vi) not having secure employment; or (vii) being from minoritised groups.

H4. There will be a difference in compassion satisfaction among mental health researchers according to the above work, health and socio-demographic factors (i-vii).

### Additional research questions

RQ1. Is there a difference in use of drugs or alcohol or sickness absence to cope with work-related feelings according to the above work, health and socio-demographic factors (i-vii)?

RQ2. Are qualitative researchers more likely to have experienced a mental health condition where they think work was a contributing factor?

RQ3. Are there gaps between perceived need for, and receipt of, workplace support for mental health researchers at HEIs?

RQ4. Is researcher well-being support currently being integrated into grant funding applications and if not, why not?

## Methods

### Participants and procedure

Academic mental health researchers across the UK were invited to complete an anonymous, 15 min, online survey between 27 March and 22 April 2024. Inclusion criteria for taking part in the research were being employed at a UK academic institution and self-identifying as working in the field of mental health research. Participants were recruited via social media and email. The study was advertised on LinkedIn and X, and via emails sent to HEIs^1^ and UK mental health research networks, including the National Institute of Health Research (NIHR) Schools for Public Health Research and Primary Care Research, GW4 Mental Health Research Network, Wellcome Mental Health and SMARTEN national network of student mental health academic researchers. A £2 donation was made on behalf of each respondent to a national mental health charity to incentivise participation. The study target sample was 200 responses based on resource and timing, and in the absence of clear population estimates to enable a power calculation. The survey was delivered using JISC software (https://www.jisc.ac.uk/data-analytics). The study was pre-registered with materials and code available on the Open Science Framework (https://osf.io/v2qwd/). Ethical approval was granted by the institution’s Health Sciences Faculty Research Ethics Committee (Ref:16453). All participants gave informed consent to taking part in the research.

## Measures

### Socio-demographics

Socio-demographic and health survey items included in the current study were region; gender; age; ethnicity; [31] sexual orientation; caring responsibilities; and disability or chronic illness. Supplementary Table S1 shows item response options and coding.

### Mental health experiences

Researchers were asked if they had currently or previously experienced a mental health condition (diagnosed or not), and/or had thoughts of self-harm or suicide. A follow-on question asked whether their work in mental health research had contributed to this, see detail in Table S1.

### Work-related characteristics

Respondents were asked about the nature of their employment and research role to include: career stage i.e. early (including PhD students), mid or senior career; nature of contract i.e., permanent or not, to reflect precarity of employment; academic discipline; research methods used i.e. qualitative or quantitative; and any experience of professional training in reflective practice or managing one’s own well-being (see Table S1).

### Secondary traumatic stress, burnout and compassion satisfaction

We used an adapted version of the Professional Quality of Life Measure (ProQOL-Version 5), a validated screen capturing the negative and positive aspects of helping others who have experienced distress or trauma [11]. It comprises three sub-scales, each with 10 items. Secondary traumatic stress (e.g., ‘As a result of my [research], I have intrusive, frightening thoughts’, *α* = 0.81) and burnout (e.g., ‘I feel worn out because of my work as a [researcher]’, *α* = 0.74) are measured alongside compassion satisfaction (e.g., ‘my work makes me feel satisfied’, *α* = 0.83). Items are scored from 1 (never) to 5 (very often), with some items reverse scored. Summed scores for each sub-scale were standardized as t-scores − 10*(( $\:\frac{(\text{}\text{Y}- \overline{\text{Y}})\:}{{\upsigma\:}\text{Y}}$) + 50) - to allow for sub-scale comparisons with all scales having mean 50 and standard deviation 10, in line with ProQOL recommendations [11]. 

### Informal coping strategies

Using adapted responses from Skinner et al.’s [21] previous findings, respondents were asked whether they had used the following informal coping strategies to manage feelings arising from their work as mental health researchers: “using drugs or alcohol” or “taking time off as sickness absence”, due to their potential negative health and economic impacts.

### Researcher workplace support

To compare types of workplace support researchers currently received with perceived need, participants were asked to select the different support they experience at work, alongside those they would like. Response items were again developed from the previous qualitative findings of Skinner and others [6, 21] e.g. projects debriefs, individual or clinical supervision, or analysing data with others (full list https://osf.io/52crb). Respondents could select all that applied.

### Funding applications

Respondents were asked about integrating researcher well-being support in funding applications. If they had submitted a mental health grant without considering researcher support, they were asked to indicate reasons for this. The list of options included: not believing funders would support the cost, or that formalised support was not necessary – see full list https://osf.io/52crb.

### Analytic strategy

Descriptive statistics (frequencies and percentages) are used to describe the survey sample and participant responses. Secondary traumatic stress, burnout and compassion satisfaction are reported in Table 1 as raw scores – means and standard deviations (SD). To analyse differences in the continuous outcomes of STS, burnout and compassion satisfaction we used simple linear regression models and report the beta co-efficients (b) and confidence intervals (95% CI). To account for multiple testing against each outcome we report a Bonferroni adjustment for the alpha threshold of 0.05 divided by the number of tests for each outcome. Results are reported in-text where they meet the Bonferroni corrected threshold of α = 0.005 unless otherwise stated.

Examination of the residuals suggested some minor deviations from the assumption of normality for STS so for models with this outcome, we report statistics using robust standard errors. Assumptions of normality were met for all other outcomes. For the categorical outcomes of factors associated with drug and alcohol use or sickness absence as a way to cope with work-related feelings, we used unadjusted logistic regression with outcomes reported as odds ratios (OR), confidence intervals (95%CI), and p values.

### Missingness and pre-processing

Respondents were unable to skip survey items, however they could select Prefer not to say. Levels of non-disclosure were low, ranging between 0 and 5.9% (see Table 1). Based on chi-squared tests, the distribution of Prefer not to say was determined to be non-systematic, therefore it was treated as missing in all further analyses.

**Table 1:** Respondent characteristics

Region, *n* (%)^a^	
Wales	18 (7.1)
East Midlands	8 (3.1)
East of England	8 (3.1)
London	45 (17.7)
North East & Cumbria	9 (3.5)
North West England	26 (10.2)
Northern Ireland	7 (2.8)
Scotland	23 (9.1)
South East England	20 (7.9)
South West England	55 (21.7)
West Midlands	5 (2)
Yorkshire and the Humber	29 (11.4)
Prefer not to say	1 (0.4)
**Gender**,** n (%)**	
Female	204 (80.3)
Male	42 (16.5)
Non-binary/Another gender	5 (2.0)
Prefer not to say	3 (1.2)
**Age in years**,** n (%)**^a^	
18–34	114 (44.9)
35–54	118 (46.5)
55 and over	21 (8.3)
Prefer not to say	1 (0.4)
**Ethnicity, n (%)**^**a**^	
Asian/Asian British	12 (4.7)
Black/Black British/Caribbean/African	3 (1.2)
Mixed or multiple ethnic groups	6 (2.4)
White	228 (90.0)
Other	3 (1.2)
Prefer not to say	2 (0.8)
**Sexual orientation**,** n (%)**	
LGBTQIA+	59 (23.2)
Straight or Heterosexual	180 (70.9)
Prefer not to say	15 (5.9)
**Caring responsibilities**,** n (%)**	
Yes	91 (35.8)
No	161 (63.4)
Prefer not to say	2 (0.8)
**Physical disability or chronic illness**,** n (%)**	
Yes	53 (20.9)
No	197 (77.6)
Prefer not to say	4 (1.6)
**Previous mental health condition (MH)**,** n (%)**	
Yes (diagnosed)	118 (46.5)
Yes (not diagnosed)	47 (18.5)
No	74 (29.1)
Not sure	13 (5.1)
Prefer not to say	2 (0.8)
**Work contributed to MH condition**,** n (%)**^a^	
Yes	29 (17.6)
No	115 (69.7)
Not sure	21 (12.7)
**Thoughts of suicide or self-harm (SSH)**,** n (%)**^a^	
Yes	101 (39.8)
No	141 (55.5)
Not sure	6 (2.4)
Prefer not say	6 (2.4)
**Work contributed to thoughts of SSH**,** n (%)**^a^	
Yes	16 (15.8)
No	81 (80.2)
Not sure	4 (3.6)
**Career stage**,** n (%)**	
Early career (inc PhD students)	154 (60.6)
Mid-career	66 (26.0)
Senior	33 (13.0)
Prefer not to say	1 (0.4)
**Permanent contract**,** n (%)**	
Yes	93 (36.6)
No	158 (62.2)
Not sure	2 (0.8)
Prefer not to say	1 (0.4)
**Academic discipline**,** n (%)**^a^	
Medicine and allied subjects	115 (45.3)
Social Sciences and psychology	116 (45.7)
Physical and biological sciences	9 (3.5)
Other e.g. law, geography, history, education	14 (5.5)
**Research methods used**,** n (%)**	
Qualitative	219 (86.2)
Quantitative (and no qualitative)	35 (13.8)
**Professional training in reflective practice/managing well-being**,** n (%)**	
Yes	78 (30.7)
No	174 (68.5)
Prefer not to say	2 (0.8)
**Professional Quality of Life Scale - raw scores**,** mean (SD)**,** range**	
Secondary traumatic stress	19.1 (5.9), 10–42
Burnout	24.6 (5.5), 10–38
Compassion satisfaction	38.1 (5.2), 23–50

^a^Reported in sample description (Table S1) but not used in analysis.

We planned to exclude ProQOL responses with the same answer for all questions but found no instances of this. In contrast to our pre-registration, we used complete case analysis (*n* = 211/254) rather than multiple imputation, because Prefer not to say was considered non-systematic. Any further deviations from our pre-registration are outlined in Supplementary material.

### Software

Stata 17 was used for all re-coding, data cleaning and analyses, including production of summary statistics. Microsoft Excel (v2406) was used to produce graphs of the data.

### Reflexivity

We (the authors) are white, female, early career researchers (aged between 30 and 55 years) working in a UK university, with a breadth of lived experience and caring responsibilities. We did not participate in the study ourselves. Our motivations for this research and selection of measures and analyses reflect our experiences as early career researchers working on topics including self-harm and suicide, anxiety, depression, and well-being. In addition, two authors have several years of experience working in social care practice and research. We identified with several of the themes described in existing qualitative literature involving trauma researchers, and with the constructs measured by the ProQOL scale, including the focus on positive aspects of compassion satisfaction as well as the negative aspects of STS and burnout.

## Results

### Sample characteristics

The survey was completed by *n* = 254 mental health researchers working across all regions of the UK. The majority of respondents were female (80.3%) and white ethnicity (90.0%), with approximately one in four identifying as LGBTQIA+ (23.2%). One in five reported a disability or chronic illness (20.9%), and a third had caring responsibilities (35.8%). The target population is ill-defined, but these sample characteristics are likely to be an overrepresentation compared to UK Research and Innovation (UKRI) or general population figures [32, 33]. 

Two thirds (65.0%) of respondents had experienced a mental health condition, with one in five (17.6%) saying work was a contributing factor. Four in ten (39.8%) had experienced thoughts of self-harm or suicide with one in six of these (15.8%) suggesting work was a contributing factor. Almost two thirds (60.6%) of respondents were early career researchers, a third (36.6%) were on a permanent contract, and one in eight (13.8%) researchers had no experience of using qualitative methods i.e. only used quantitative methods. Less than a third (30.7%) of respondents had experienced any professional training in reflective practice or managing their own well-being. Table 1 shows detailed sample characteristics and distribution of non-disclosure; Table S2 provides a correlation matrix between these outcomes as binary variables.

### Professional quality of life - Secondary traumatic stress, Burnout and Compassion satisfaction (Hypotheses 1–4)

Mean scores and standard deviations of secondary traumatic stress, burnout and compassion satisfaction are shown in Table 1. Outcomes of each potential factor on the three measured sub-types of researcher professional quality of life are shown in Table 2. Contrary to our confirmatory hypothesis (H1) we found no evidence to support an association between career stage and STS scores. However, there was evidence that using qualitative research methods (compared to only quantitative methods) was associated with increased levels of STS (*b* = 6.07, 95%CI = 2.91; 9.22, t = 3.79, *p* < .001). We also found that having personal lived experience of a mental health condition was associated with increased levels of STS (*b* = 6.38, 95%CI = 3.74; 9.02, t = 4.76, *p* < .001). Having a chronic illness or disability (*b* = 5.87, 95%CI = 2.20; 9.53, t = 3.15, *p* < .01), but not caring responsibilities, was also associated with increased levels of STS. There was no evidence that those without permanent employment contracts had increased levels of STS compared to those with permanent roles. We found no differences in gender or LBGTQIA + status and were unable to test ethnicity given the small numbers of respondents from minoritised ethnic backgrounds.

**Table 2:** Factors associated with secondary traumatic stress, burnout, and compassion satisfaction

	Secondary traumatic stress				Burnout				Compassion satisfaction			
	b	95%CI	t-test	*p*-value	b	95%CI	t-test	p-value	b	95%CI	t-test	*p*-value
**Career stage**												
Mid/late career	(ref)				(ref)				(ref)			
Early career	2.25	−0.46; 4.97	1.64	0.103	−1.00	−3.77; 1.77	− 0.71	0.477	− 0.47	−3.24; 2.30	− 0.33	0.739
**Qualitative research involvement**												
No	(ref)				(ref)				(ref)			
Yes	6.07	2.91; 9.22	3.79	0.000**	4.98	1.03; 8.93	2.48	0.014*	− 0.33	4.34; 3.68	− 0.16	0.872
**Personal experience of mental health condition**												
No	(ref)				(ref)				(ref)			
Yes	6.38	3.74; 9.02	4.76	0.000**	7.32	4.47; 10.17	5.07	0.000**	−5.66	−8.56; −2.77	−3.85	0.000**
**Physical disability or chronic illness**												
No	(ref)				(ref)				(ref)			
Yes	5.87	2.20; 9.53	3.15	0.002**	5.62	2.39; 8.84	3.43	0.001**	−1.36	−4.47; 1.95	− 0.81	0.419
**Caring responsibilities**												
No	(ref)				(ref)				(ref)			
Yes	0.55	−2.33; 3.42	0.37	0.709	− 0.38	−3.21; 2.45	− 0.27	0.790	− 0.65	−3.48; 2.17	− 0.46	0.648
**Professional training in reflective practice/managing well-being**												
Yes	(ref)				(ref)				(ref)			
No	0.83	−2.14; 3.79	0.55	0.583	2.62	− 0.26; 5.51	1.79	0.0751	−4.34	−7.19; −1.50	−3.01	0.003**
**Permanent contract**												
Permanent	(ref)				(ref)				(ref)			
Not permanent/Unsure	0.80	−1.97; 3.58	0.57	0.568	−0.23	−3.04;2.58	−0.16	0.871	−2.89	−5.67; −0.11	−2.05	0.042*
**Gender**												
Female^a^	(ref)				(ref)				(ref)			
Male	−1.69	−5.37; 1.99	−0.91	0.366	1.09	−2.69; 4.87	0.57	0.570	− 0.46	−4.22; 3.31	− 0.24	0.811
**Sexual orientation**												
Heterosexual	(ref)				(ref)				(ref)			
LGBTQIA+	3.32	−0.04; 7.02	1.77	0.078	2.32	− 0.90; 5.54	1.42	0.156	−3.15	−6.34; 0.04	−1.94	0.053

*0.05 ** 0.005 Bonferroni threshold

^a^Non- binary gender was not used in this analyses due to small group size

Contrary to our confirmatory hypothesis (H2), we found no evidence that researchers with professional training in managing well-being had lower levels of STS than those without training (see Table 2).

With regard to burnout (H3), there was some evidence that using qualitative methods was associated with higher levels of burnout compared to researchers using only quantitative methods (*b* = 4.98, 1.03; 8.93, *p* = .014) but this did not meet the Bonferroni threshold. Having a mental health condition (*b* = 6.38, 3.55; 9.22, *p* < .001) or a physical disability or chronic illness (*b* = 5.87, 2.60; 9.13, *p* < .001) was more clearly associated with higher levels of burnout. There were no further differences according to work, health or socio-demographic factors.

In relation to compassion satisfaction (H4), researchers with personal experience of a mental health condition reported lower compassion satisfaction than those without (*b*=−5.66, 8.56; −2.77, *p* < .001). There was also some evidence of lower compassion satisfaction for those who were not on a permanent employment contract but this did not meet the Bonferroni threshold (*b=*−2.89, −5.67; −0.11, *p* = .042). The only other differences in researcher characteristics were seen in lower compassion satisfaction for researchers without any professional training in managing well-being (*b*= −4.34, −7.19; −1.50, *p* = .003).

### Informal coping strategies (RQ1)

A quarter of respondents (25.2%) reported using drugs or alcohol to cope with feelings related to their work in mental health research, and one in five (21.2%) had taken time off work as sickness leave. Table 3 shows odds ratios for each of the researcher characteristics in relation to coping strategies. Researchers with a mental health condition had more than twice the odds of using drugs or alcohol to cope with work-related feelings compared to those without a mental health condition, though this association did not reach the Bonferroni threshold (OR = 2.12, 95%CI: 1.05; 4.28, *p* = .036). Researchers identifying as male also had more than twice the odds of using drugs or alcohol to cope compared to those who identified as female, but this again did not meet the Bonferroni threshold (OR = 2.40, 95%CI:1.19; 4.84, *p* = .014).

**Table 3:** Coping with feelings related to mental health research

	Use of drugs/alcohol			Taking sickness absence/ leave		
	OR	95%CI	p-value	OR	95%CI	p-value
**Career status**						
Mid/late career	(ref)			(ref)		
Early career	0.63	0.35; 1.12	0.113	1.47	0.77; 2.80	0.238
**Qualitative research involvement**						
No	(ref)			(ref)		
Yes	1.41	0.58; 3.40	0.447	1.73	0.64; 4.69	0.282
**Personal lived experience of mental health condition**						
No	(ref)			(ref)		
Yes	2.12	1.05; 4.28	0.036*	10.00	3.00; 33.28	0.000**
**Physical disability or chronic illness**						
No	(ref)			(ref)		
Yes	1.26	0.64; 2.49	0.507	1.50	0.74; 3.04	0.259
**Caring responsibilities**						
No	(ref)			(ref)		
Yes	0.99	0.55; 1.79	0.973	0.55	0.28; 1.08	0.080
**Professional training**						
Yes	(ref)			(ref)		
No	1.48	0.78; 2.81	0.235	1.21	0.62; 2.36	0.570
**Permanent contract**						
Permanent	(ref)					
Not permanent/Unsure	0.96	0.53; 1.72	0.887	1.21	0.64; 2.28	0.556
**Gender**						
Female	(ref)			(ref)		
Male	2.40	1.19;4.84	0.014*	1.33	0.62; 2.86	0.467
**Sexual orientation**						
Heterosexual	(ref)			(ref)		
LGBTQIA+	1.40	0.73; 2.68	0.313	2.12	1.09; 4.11	0.027*

*0.05 ** 0.005 Bonferroni threshold

Researchers with personal lived experience of a mental health condition had ten times higher odds of taking sickness absence to cope with work-related feelings compared to those without a mental health condition (OR = 10.00, 95%CI: 3.00; 33.28, *p* < 001). Researchers identifying as LGBTQIA + had twice the odds of taking sickness absence compared to those who did not identify as LGBTQIA+, but this did not reach the Bonferroni threshold (OR = 2.12, 95%CI:1.09;4.11, *p* = .027). We found no evidence of further differences in use of drugs and alcohol or sickness absence to cope with work-related feelings according to work, health or socio-demographic factors.

We were unable to test (RQ2) whether researchers using qualitative methods were more likely to have a mental health condition they think was caused by their work (compared to only using quantitative methods) due to small cell counts.

### Researcher workplace support (RQ3)

**Fig. 1:**
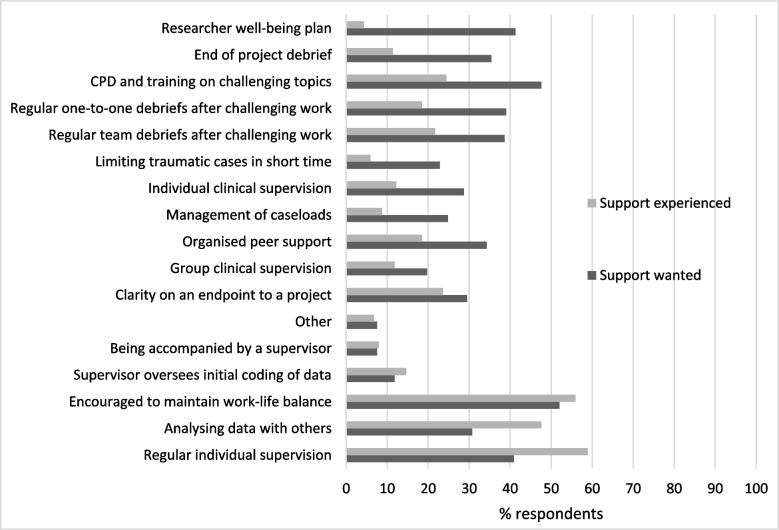
Ranked differences between % researchers experiencing and wanting types of workplace support

The most commonly experienced types of workplace support for mental health researchers were regular individual supervision i.e. with a supervisor (58.9%); being encouraged to take leave and maintain work life balance (55.9%); and analysing data with others (47.6%). Figure 1 shows the biggest differences between perceived need and having experience of workplace support. The largest discrepancies related to researcher well-being plans, where a small proportion of respondents had developed them in the past (4.3%), but with many more wanting them (41.3%). Similar differences were seen for end of project debriefs, with fewer experiencing them (11.4%) compared to wanting them (35.4%); continuing professional development and training on challenging topics (where 24.4% had experienced, and 47.6% wanted); regular one to one debriefs (18.5%, 39.0%); team debriefs (21.7%, 38.6%); limiting time on traumatic cases (5.9%, 22.8%); and clinical supervision (12.8%, 22.8%).

### Funding applications (RQ4)

More than two thirds of respondents had experience of writing a mental health grant (*n* = 177/254; 70.7%), but, of these, the majority had never costed researcher well-being support into an application (*n* = 154/177; 87.0%). For those who had never costed support into an application (*n* = 154) the biggest reason for not doing so, was not having considered it as an option (63.6%). Only a small proportion indicated they did not think it was necessary (4.6%). Almost half of these respondents believed funders would not support the cost (43.5%) with others not knowing what types of support to provide (36.4%), or who would provide or organise it (44.2%).

## Discussion

Our study offers the first quantitative evidence for how the professional quality of life of academic mental health researchers might be impacted by their work. Attention to date has been focused on supporting clinicians and frontline professionals working in caring professions, and more recently researchers working on challenging topics such as conflict, trauma or abuse [21, 28]. However, our novel findings suggest UK mental health researchers may have similar if not higher than average secondary trauma and burnout scores compared to some of their frontline clinical colleagues [27, 28]. Rates of STS and burnout in our respondents match those recently seen in psychologists working in New Zealand during the Covid-19 pandemic [28] and are significantly higher than those seen in front-line Italian hospital staff in 2020 at the pandemic peak [27]. 

Critically, our study also highlights important socio-demographic, health, and work-related differences in the mental health experience and professional quality of life of the UK mental health researchers we surveyed. Two thirds of respondents reported personal lived experience of a mental health condition, which perhaps not surprisingly, showed the strongest association with poorer professional quality of life for researchers i.e. higher levels of secondary traumatic stress and burnout and lower compassion satisfaction. This is supported by previous research suggesting that having a mental health condition would likely be negatively associated with most measurements of quality of life [13, 14, 19, 23, 26–28]. Almost one in five of those who reported mental health challenges also described work as a contributing factor, which has critical implications for employers recruiting researchers to mental health projects, particularly where experiential expertise is pro-actively sought [9]. 

Similarly, the finding that qualitative researchers show poorer professional quality of life is consistent not only with our expectations but offers new statistical evidence supporting previous work [5, 6, 12, 21, 22]. It suggests secondary trauma and burnout may be heightened among those exposed to detailed information concerning individual stories and graphic content or through cumulative exposure to emotionally challenging fieldwork. Existing research has suggested this vulnerability for qualitative researchers may relate to increased empathising or identifying with participants, rumination on safeguarding issues, and a feeling of powerlessness to improve another individual’s situation [12, 21, 22, 29]. For academic mental health researchers, many of whom work remotely post Covid-19, this feeling may be heightened by working alone or remotely [34]. 

Our findings also provide some support for qualitative insights from previous research that researchers from marginalised communities may be more at risk of secondary trauma and burnout related to work, in particular those with chronic illness or a disability [7]. This adds a professional quality of life dimension to an already established literature linking disability and mental health outcomes [35] and highlights a further work-related vulnerability for this group. Contrary to our expectations based on previous research [36], we did not find evidence of poorer outcomes among early career stage researchers. However, that is echoed in findings from the New Zealand psychologists study [28] where the authors found greater compassion fatigue in those with more years’ experience, and hypothesised a survivor effect, where early career professionals leave the field if experiencing burnout. Fenge et al. [37] suggest more experienced researchers may encounter unexpected emotional responses to specific research projects in the same way as more junior colleagues, and could benefit from reflections around their own professional quality of life. Others [12, 21] have also suggested that senior researchers may be vulnerable to cumulative effects of exposure to secondary traumatic stress as well as the additional burden of responsibility that comes with managing or supervising others.

With regard to compassion satisfaction, our findings show lower levels for researchers who either have personal mental health experience, are on insecure contracts, or have no formal training in managing their own well-being, which also supports previous insights from qualitative work such as the Researcher Wellbeing Project [21]. Our results suggest that the positive aspects of professional quality of life in helping and caring professions, like job fulfilment and reward, may be undermined by a lack of work-place support or employment security. Employment insecurity is a well-documented source of poor mental health and well-being among academic researchers [24]. Whilst our study focuses on researcher mental health and professional quality of life relating specifically to mental health-related topics, these issues are likely compounded by the stress of contract precarity. Additionally, contract precarity can result in short-term recruitment onto mental health projects for researchers from other fields with little experience or preparation for the accompanying potential emotional challenges.

Our findings also extend previous work [13, 21, 22] which highlight some key differences in how researchers report managing their own feelings arising from their research and what formal and informal support might be available or desirable to them. Having lived experience of a mental health condition was associated with both using drugs or alcohol to cope and sick leave in our study. Qualitative research with researchers working on gender-based violence topics suggested that some researchers used alcohol as a means of coping with secondary traumatic stress [12]. In McKenzie et al., [22] interviews with research assistants working on suicide-related topics revealed the importance of flexibility in work patterns in terms of knowing ‘when to stop and walk away’ from data collection. These findings underline the importance of providing adequate support to researchers with previous lived experience of a mental health condition – including a strong economic argument for avoiding unplanned absence in the HEI workforce [30]. Our results also suggest that identifying as male was associated with increased likelihood of using drugs or alcohol to cope with work-related stress and identifying as LGBTQIA + was associated with increased likelihood of taking sick leave to cope. This is a novel finding with no comparative evidence, but if it were also found in human resource data from HEIs, institutions may want to focus on intervention for these specific groups.

Despite predicting otherwise, our study offers only limited evidence that professional training in managing one’s own well-being is associated with better professional quality of life. However, almost half of survey respondents indicated that they would like to receive CPD or training in handling challenging research topics. A substantial number of researchers wanted a researcher well-being plan, while very few had actually received them. This strengthens calls elsewhere for personalised researcher toolkits for coping strategies and support – an important factor in being prepared for the emotional impact of research [7, 12, 21, 22]. 

The demand for workplace support identified in our study is also consistent with qualitative findings from Fenge et al. [37], who found a lack of preparedness among social science researchers engaged in emotionally demanding qualitative research. Training and support for reflecting on self-care requirements during such research was highlighted in their research as a key area for improvement to enhance researcher resilience during challenging periods of research. For experienced researchers in particular, external supervision was recommended to provide a safe reflective space in which to develop reflexivity [37]. In our study, clinical supervision was endorsed by almost a quarter of researchers, highlighting this as a potential cost to consider including in funding applications [21]. Regular individual and team debriefs during (and at the end of) an emotionally challenging project were endorsed by two in five researchers in our study; it was one of the largest mismatches between support experienced and wanted by mental health researchers. Further findings from Fenge et al. [37] underpin this with researchers raising the issue of positionality and identity as key to developing resilience, suggesting that reflective and reflexive conversation with peers and supervisors could be an important avenue for support. This type of support creates an opportunity not only to exchange ideas for effective coping strategies but also to normalise emotional responses to research while promoting collegiality and unity among researchers [15, 37]. 

Our study also reveals that, though small, a number of researchers report experience of a mental health condition and/or thoughts of self-harm or suicide where their work in mental health research was felt to be a contributing factor. Serious consideration – encouraged through ethics committees and grant funding bodies – must be given to adequate assessment of psychological risk to researchers working on mental health topics in the same way it is under the Helsinki agreement for research participants [7, 21, 22, 38]. Our findings show that although very few researchers consider researcher mental health and well-being when writing grant and funding applications, it is not because they do not consider it important, but because they do not feel equipped or informed enough to do so.

### Strengths and limitations

This study offers novel quantitative evidence on the epidemiology of professional quality of life among mental health researchers. It extends and complements existing and ongoing qualitative research work in mental health research and other emotionally challenging topics. It examines researcher characteristics in detail, such as sociodemographic differences, personal mental health experience and work-related factors including types of research methodologies used and different career stages, and in relation to professional quality of life. It also offers key quantitative evidence in the UK sector for what support HEI researchers have experienced versus the type of support they would like to have.

Our findings should also be viewed in light of several limitations. First, we used convenience sampling to obtain survey respondents, which is likely to result in overrepresentation of researchers experiencing work-related mental health challenges – who may be more interested in the study – and underrepresentation of those outside the fields of psychology and public health. With no objective description of the target population’s socio-demographics, we are unable to draw conclusions on the representativeness of our sample, and subsequent generalisability of our findings, compared to UK academic mental health researchers as a whole. Second, our study will not have captured the experiences of those researchers who have left academia as a result of trauma, burnout or stress, leading to potential survivor bias within the sample. Third, we are unable to separate out associations with burnout relating specifically to work in mental health research rather than academia in general. Issues such as employment precarity are likely to impact researchers beyond the implications of working on emotionally challenging topics. Fourth, although the ProQOL instrument is the most widely used measure of professional quality of life in helping and caring professions, and had good internal consistency in our sample, some concerns have been raised regarding its validity, dependent on occupational context [39]. Further research will be needed to establish the scale’s validity in academic researcher populations. Similarly, a number of our survey items were pragmatically designed to quantify challenges experienced by researchers highlighted by previous research, such as ways of coping with work-related stress. While these items extend previous qualitative findings and offer preliminary quantitative evidence for how researchers might experience the impact of their work, we recognise the limitations of using previously unvalidated measures. Finally, we used statistical correction methods i.e. Bonferroni and robust standard error adjustments, to account for the number of tests conducted. Where we report weak results that do not meet the thresholds, there may have been insufficient power in our sample to detect smaller effects. This could have been a result of unequal group sizes within the sample for some of the variables of interest. Larger samples would be recommended in future work with this population.

### Implications

This is some of the first work to empirically investigate academic researchers’ experience of their own mental health, professional quality of life, and workplace support needs. It strengthens qualitative work like that of Skinner et al. [21] who have found similar themes on the impact of undertaking challenging research. We recommend and support adoption of standard practices for academic researchers working in mental health in the higher education sector [12]. 

While the expertise of researchers with lived experience is increasingly acknowledged and valued in mental health research [9], there is also increasing awareness of the potential harm this may cause [40]. Our findings underline the importance of providing adequate support for this group, whether or not they formally identify as lived experience researchers, to enable critical contribution of a rich multidimensional perspective to mental health research. Our results suggest that researchers with lived mental health experience may particularly benefit from targeted support. This might include recommended proactive support such as development of comprehensive well-being plans prior to undertaking a project access to relevant training, and clinical supervision where appropriate throughout a project lifecycle to address emotional responses to research. Such proactive approaches should be available in addition to reactive staff well-being services such as counselling [7, 21]. 

Those without lived mental health experience may still experience secondary trauma or be vulnerable to various emotional challenges of working closely with sensitive data or directly with participants experiencing distress, severe mental health issues or with ‘difficult’ stories to tell. Our findings additionally highlight the importance of targeting proactive support measures to researchers engaging with qualitative methods as a priority group, as well as for those with disabilities and/or chronic illness who may have added vulnerabilities. That might include management of workloads and limiting very traumatic cases researched during a short space of time; funded regular clinical supervision at least during fieldwork – the costs of which should be considered against the economic cost to HEIs of researcher burnout; [12, 21] individual or group peer supervision, ongoing professional development and training to prepare for emotionally demanding research; supporting positive work-life balance and regular leave.

Further detailed recommendations made elsewhere [21] would also be supported by our findings - they include an ethics committee mandate and researcher risk assessment for researcher self-care, including the specific needs of those working from home [7, 29]. 

## Conclusions

This study highlights the wealth of lived experience amongst those working on mental health topics, and emphasises the need for systematic, proactive support for this group. It particularly underlines the importance for researchers with additional considerations such as their own mental health challenges or lived experience, those undertaking qualitative research, and those facing disabilities and/or chronic illness.

Our findings offer new, quantitative insights that complement the growing body of qualitative evidence making essential recommendations for a range of proactive support measures to enhance work-related quality of life among academic researchers and to prevent work-related absenteeism. That includes researcher well-being plans, personal development and training, and project debriefs as standard both with supervisors and the research team as a whole. Consideration should be given to costing clinical supervision into grant applications where appropriate, and researcher well-being should be considered in funding, grant and ethics applications in the same way it is for those who take part in our research studies.

## Supplementary Information

Additional file 1.

## Data Availability

The datasets generated and analysed during the current study are available under restricted access in the data.bris Research Data Repository, 10.5523/bris.20vp1h9743fij2tbqcjppnz296.

## Footnotes

1 Emails were sent to known contacts, departmental administrators and departmental leads in health and psychology departments at HEIs across the UK, including: Universities of Bristol, Bath, Edinburgh, Glasgow, Sussex, Cambridge, Bournemouth, Liverpool, Manchester, York, Newcastle, Exeter, Swansea, Edge Hill, East Anglia, Leeds, Belfast, Southampton, Surrey, Aberdeen, Sheffield, Cardiff, Wolverhampton, University College London, Kings College London, Imperial, Nottingham, Birbeck and Oxford. To protect anonymity, we did not collect data on participants’ institution, other than region.
