# Supplementary material for: Understanding and supporting the mental health and professional quality of life of academic mental health researchers: results from a cross-sectional survey
Source: BMC Public Health. 2025 Feb 15;25:632. doi: 10.1186/s12889-025-21823-3 (PMC11829397; doi:10.1186/s12889-025-21823-3)
Supplement: Supplementary file 1 — Additional file 1. [file 12889_2025_21823_MOESM1_ESM.pdf]

Understanding and supporting the mental health and professional quality of life of academic mental health researchers: Results from a cross-sectional survey (Supplementary material)

Table S1 Study variables and coding

**Variable**	**Coding** (reference groups underlined)
Region†	Wales, East Midlands, East England, London, NE & Cumbria, NW England, N Ireland, Scotland, SE England, SW England, West Midlands, Yorkshire & Humber
Gender	Female; Male; Non-binary
Age in years†	18-34; 35-54; >55
Ethnicity*†	White; Mixed; Asian; Black; Other ethnic group
Sexual orientation	Heterosexual; LGBTQIA+ (including those who indicated they were gay or lesbian, bisexual, another sexual orientation, or whose gender was not the same as their sex assigned at birth)
Caring responsibilities	No; Yes (any)
Physical disability or chronic illness	No; Yes
Experienced a mental health (MH) condition	No; Yes (diagnosed and undiagnosed)
Work as a contributing factor to MH condition†	No; Yes (only those answering yes to above)
Thoughts of suicide or self-harm (SSH)†	No; Yes
Work as a contributing factor to thoughts of SSH†	No; Yes (only those answering yes to above)
Career stage	Mid/senior career; Early career (including PhD students)
Nature of employment contract	Permanent; Not permanent/unsure
Academic discipline†	Medicine and allied subjects; Social sciences; Physical and biological sciences; Other
Research methods used	No qualitative research; Any method of qualitative research (including conducting interviews or focus groups, transcribing interview or focus group data, reading case files, analysing qualitative data, analysing open text data or ethnography)
Professional training in reflective practice/ managing well-being	Yes; No

† Reported in sample description (Table 1) but not used in analysis
*Categories according to ONS, 2021.(31)

Table S2. Pearson correlation matrix of binary variables used in Table 2 analyses.

	Career stage	Qualitative research involvement	Personal experience of MH condition	Physical disability or chronic illness	Caring responsibilities	Professional training in reflective practice/managing well-being	Permanent contract	Gender	Sexual orientation
Career stage	1.000								
Qualitative research involvement	-0.079	1.000							
Personal experience of MH condition	0.112	0.015	1.000						
Physical disability or chronic illness	-0.080	-0.026	-0.162	1.000					
Caring responsibilities	0.356	-0.080	0.050	-0.132	1.000				
Professional training in reflective practice/ managing well-being	0.070	-0.143	0.054	-0.054	0.054	1.000			
Permanent contract	0.403	0.053	0.050	-0.072	0.258	0.153	1.000		
Gender	-0.025	-0.009	-0.025	0.081	0.135	-0.007	-0.124	1.000	
Sexual orientation	0.254	-0.020	0.154	-0.132	0.243	0.136	0.103	0.155	1.000

Deviations from pre-registration:

We did not analyse motivational differences in researchers with high secondary traumatic stress or compassion satisfaction scores due to the high correlation with personal experience of a mental health condition.

We did not conduct further analysis on whether qualitative researchers were more likely to have a mental health condition caused by their work due to small sample numbers.
